# ADHD/CD-NET: automated EEG-based characterization of ADHD and CD using explainable deep neural network technique

**DOI:** 10.1007/s11571-023-10028-2

**Published:** 2023-11-28

**Authors:** Hui Wen Loh, Chui Ping Ooi, Shu Lih Oh, Prabal Datta Barua, Yi Ren Tan, U. Rajendra Acharya, Daniel Shuen Sheng Fung

**Affiliations:** 1 School of Science and Technology, Singapore University of Social Sciences, Singapore, Singapore; 2 Cogninet Australia, Sydney, NSW 2010 Australia; 3 Faculty of Engineering and Information Technology, University of Technology Sydney, Sydney, NSW 2007 Australia; 4 School of Business (Information System), University of Southern Queensland, Darling Heights, Australia; 5 Australian International Institute of Higher Education, Sydney, NSW 2000 Australia; 6 School of Science & Technology, University of New England, Armidale, Australia; 7 School of Biosciences, Taylor’s University, Selangor, Malaysia; 8 School of Computing, SRM Institute of Science and Technology, Kattankulathur, India; 9 School of Science and Technology, Kumamoto University, Kumamoto, Japan; 10 Sydney School of Education and Social work, University of Sydney, Camperdown, Australia; 11 Developmental Psychiatry, Institute of Mental Health, Singapore, Singapore; 12 School of Business (Information Systems), Faculty of Business, Education, Law & Arts, University of Southern Queensland, Darling Heights, Australia; 13 School of Mathematics, Physics and Computing, University of Southern Queensland, Springfield, Australia; 14 Centre for Health Research, University of Southern Queensland, Springfield, Australia; 15 Lee Kong Chian School of Medicine, DUKE NUS Medical School, Yong Loo Lin School of Medicine, Nanyang Technological University, National University of Singapore, Singapore, Singapore

**Keywords:** Explainable artificial intelligence (XAI), Deep learning, ADHD, Conduct disorder, Grad-CAM, CNN, EEG

## Abstract

In this study, attention deficit hyperactivity disorder (ADHD), a childhood neurodevelopmental disorder, is being studied alongside its comorbidity, conduct disorder (CD), a behavioral disorder. Because ADHD and CD share commonalities, distinguishing them is difficult, thus increasing the risk of misdiagnosis. It is crucial that these two conditions are not mistakenly identified as the same because the treatment plan varies depending on whether the patient has CD or ADHD. Hence, this study proposes an electroencephalogram (EEG)-based deep learning system known as ADHD/CD-NET that is capable of objectively distinguishing ADHD, ADHD + CD, and CD. The 12-channel EEG signals were first segmented and converted into channel-wise continuous wavelet transform (CWT) correlation matrices. The resulting matrices were then used to train the convolutional neural network (CNN) model, and the model’s performance was evaluated using 10-fold cross-validation. Gradient-weighted class activation mapping (Grad-CAM) was also used to provide explanations for the prediction result made by the ‘black box’ CNN model. Internal private dataset (45 ADHD, 62 ADHD + CD and 16 CD) and external public dataset (61 ADHD and 60 healthy controls) were used to evaluate ADHD/CD-NET. As a result, ADHD/CD-NET achieved classification accuracy, sensitivity, specificity, and precision of 93.70%, 90.83%, 95.35% and 91.85% for the internal evaluation, and 98.19%, 98.36%, 98.03% and 98.06% for the external evaluation. Grad-CAM also identified significant channels that contributed to the diagnosis outcome. Therefore, ADHD/CD-NET can perform temporal localization and choose significant EEG channels for diagnosis, thus providing objective analysis for mental health professionals and clinicians to consider when making a diagnosis.

**Supplementary Information:**

The online version contains supplementary material available at 10.1007/s11571-023-10028-2.

## Introduction

Attention deficit hyperactivity disorder (ADHD) is a common pediatric neurodevelopment disorder, with a global prevalence of 5% among people aged 18 and under (Sayal et al. 2018). Due to the lack of dopamine production, the prefrontal cortex, which is crucial for managing behavior, emotion, and attention, is particularly undeveloped in those with ADHD (Arnsten 2009; Loh et al. 2022a; Barua et al. 2022). As such, ADHD individuals exhibit forgetfulness, disorganization, and loss of concentration and attention (Magnus et al. 2021). Hence stimulant medications like Ritalin or Concerta, which can increase dopamine levels in the brain, are frequently used to treat ADHD (Arnsten 2009). If diagnosed early and treated promptly, a recovered individual may be able to restore neuronal connections to the prefrontal cortex and resume normal activities of daily living (Mattfeld et al. 2014). Otherwise, ADHD symptoms may persist into adulthood, increasing the likelihood of developing depression and antisocial behaviors as well as other undesirable outcomes like crime, academic underachievement, interpersonal relationship issues, and low employability (Sayal et al. 2018; Shaw et al. 2012; TAYLOR et al. 1996). Conduct disorder (CD) is frequently comorbid in approximately 30% of ADHD cases (Biederman et al. 1991). Some characteristics of CD include aggression towards people and animals, theft, violation of rules, and destruction of properties (Lillig 2018b). Furthermore, it has been revealed that patients with ADHD and CD are the least responsive to treatment (Carpentier et al. 2012; Shaw et al. 2012). Therefore, we must identify ADHD patients who are comorbid with CD to arrange a different treatment protocol that is best suited to them rather than receiving the same treatment procedure as ADHD.

According to the American Psychiatric Association, a comprehensive clinical interview and behavior rating scales are required to confirm a diagnosis of ADHD or CD (Levy 2014; Marshall et al. 2021; Salekin 2016). Clinical interviews are conducted with patients, family members, and teachers to determine if the patients exhibit symptoms in multiple settings (Marshall et al. 2021; Valo and Tannock 2010). Behavioral rating scales are frequently used in conjunction with clinical interviews, and they are designed to meet the Diagnostic and Statistical Manual of Mental Disorders (DSM) diagnostic requirements (American Psychiatric Association 2013; Marshall et al. 2021). These assessment approaches, however, are based on subjective judgments, and the evaluation process is lengthy and tedious, which delays the diagnosis and impedes the delivery of the timely intervention. Furthermore, subjective evaluation of symptoms is prone to misdiagnosis (Sansone and Sansone 2011); there have been cases where students mimic ADHD symptoms to obtain ADHD prescription stimulants, which can aid in concentration (Hall et al. 2005), weight management (Piran and Robinson 2006), academic (Rabiner et al. 2010) and athletic performance (McDuff and Baron 2005). Therefore, an objective evaluation of ADHD and CD is essential in facilitating early diagnosis and reducing the likelihood of misdiagnosis and abuse of ADHD prescription stimulants.

Studies have shown visible differences in brain activities recorded using electroencephalograms (EEG) and magnetic resonance imaging (MRI) between ADHD and controls (Sridhar et al. 2017; Travell and Visser 2006), non-medicated responders and medicated responders (Loo and Barkley 2005). This study will be using EEG data acquired from ADHD, ADHD + CD and CD patients to develop a computer-aided diagnostic (CAD) tool based on artificial intelligence. As EEG is high-dimensional data with a number of features exceeding that of observations, we will be using a deep learning (DL) model, convolutional neural network (CNN) in particular, rather than the conventional machine learning (ML) models to perform the classification (Mirza et al. 2019). This is because feature extraction and selection—are crucial procedures when creating a ML model—can result in information loss (Faust et al. 2019; Loh et al. 2020; Mirza et al. 2019). DL models based on neural networks, can process high-dimensional data with minimal information loss (Faust et al. 2019). Additionally, the feature extraction and selection process is not necessary to develop a DL model (Faust et al. 2019).

Hence, we propose ADHD/CD-NET, a DL system that is a cost-effective CAD tool for ADHD and CD diagnosis. DL models, however, are not without drawbacks. The ‘black box’ nature of the DL model results in poor interpretability of the results, as neither the clinicians nor developers have information on how the DL model comes about with its prediction (Loh et al. 2022). Fortunately, explainable artificial intelligence (XAI) techniques have recently been developed to provide explanations for the DL model’s predicted results (Barredo Arrieta et al. 2020; Nazar et al. 2021). In this study, ADHD/CD-NET incorporates a well-known XAI technique known as gradient-weighted class activation mapping (Grad-CAM) to provide an interpretation of the predicted result (Zhou et al. 2015). The novelties of our study are summarized as follows:

- To the best of our knowledge, this is the first study to use explainable DL approaches to analyze EEG data to distinguish ADHD from its comorbidities, ADHD + CD and CD.
- We have also proposed a unique EEG preprocessing strategy, which involves estimating the Pearson correlation coefficient between EEG channels and then transforming the correlated EEG channels using CWT to generate a channel-wise CWT correlation matrix.
- We employed the XAI technique (Grad-CAM) to visualize the interactions between EEG channels for patients with ADHD, ADHD + CD, and CD. This technique highlighted significant pairs of correlated EEG channels that played a crucial role in the classification of ADHD, ADHD + CD, and CD patients.

## Related works

Numerous studies have been conducted to detect ADHD objectively. Table 1 contains a list of recent works completed between 2017 and 2022 that focused on distinguishing ADHD patients from healthy controls using EEG signals. Exceptional performance have been achieved with ML and DL models, with the lowest classification accuracy being 81% (Kim et al. 2021) and 83% (Vahid et al. 2019) for ML and DL models, respectively, and the highest being 100% (Kaur et al. 2020; Öztoprak et al. 2017), and 99.50% (Ahmadi et al. 2021). In addition, 7 out of 9 DL studies proposed using the CNN model, demonstrating that the CNN model is the go-to DL model for EEG analysis in ADHD detection, which we had also adopted in our study. Previous works have successfully demonstrated that the EEG characteristics of ADHD patients differ from those of healthy controls. The next step in improving ADHD diagnosis will be to further distinguish those diagnosed with ADHD from CD, which is a common comorbidity of ADHD that is frequently misdiagnosed as the other due to similar clinical symptoms (FARAONE et al. 1997; KUHNE et al., 1997). Hence distinguishing ADHD, ADHD + CD, and CD is a much more difficult task compared to ADHD and healthy controls. Therefore, this study proposed ADHD/CD-NET, a deep learning system for objectively identifying ADHD from CD using EEG signals.

**Table 1:** List of related EEG-based ML and DL studies that used the ADHD vs. healthy control dataset to detect ADHD

Study	Subjects	Sampling Frequency	Features	Classifier	Accuracy (%)
Deep learning (DL)					
(Zhou et al. 2022)			End-to-end EEG	CNN	97.70
(Wang et al. 2022)	44 HC 52 ADD 48 ADHD	256 Hz	Event-related potential (ERP)	CNN-LSTM	98.23
(Tosun 2021)	1088 HC 1088 ADHD	500 Hz	Power spectral features	LSTM	92.20
(Ahmadi et al. 2021)	14 HC 13 ADHD-C 12 ADHD-I	250 Hz	Power spectral features	CNN	99.50
(Dubreuil-Vall et al. 2020),	20 HC 20 ADHD	500 Hz	Spectrograms	CNN	88.00
(Moghaddari et al. 2020),	30 HC 31 ADHD	128 Hz	Powers pectral band separation Making RGB images	CNN	98.50
(Vahid et al. 2019)	44 HC 48 ADHD	500 Hz	End-to-end EEG	CNN	83.00
(Chen et al. 2019b)	57 HC 50 ADHD	1000 Hz	Power spectral features	CNN	90.30
(Chen et al. 2019a)	51 HC 50 ADHD	1000 Hz	Connectivity matrix	CNN	94.70
Machine learning (ML)					
(Barua et al. 2022)	60 HC 61 ADHD	128 Hz	Ternary motif pattern-based features	kNN	95.57
(Kim et al. 2021)	45 HC 34 ADHD	1000 Hz	MMN source activity features	SVM	81.00
(Guney et al. 2021)	38 HC 27 ADHD	1000 Hz	Event-related potential (ERP)	ANN	98.40
(Catherine Joy et al. 2022)	5 HC 5 ADHD	256 Hz	Power spectral features	ANN	99.80
(Altınkaynak et al. 2020)	23 HC 23 ADHD	2500 Hz	Morphologi cal, nonlinear, and wavelet features	MLP	91.30
(Rezaeezadeh et al. 2020)	12 HC 12 ADHD	256 Hz	Power spectral features	SVM	99.60
(Müller et al. 2020)	147 HC 181 ADHD	500 Hz	Power spectral features, ERP peak amplitudes and latencies	SVM	80.00
(Chen et al. 2019a, b, c)	58 HC 50 ADHD	1000 Hz	Power spectral features	SVM	84.60
(Kaur et al. 2020)	50 HC 47 ADHD	256 Hz	PSR-PSO	NDC	100
(Khoshnoud et al. 2018)	12 HC 12 ADHD	256 Hz	Power spectral features	SVM	83.30
(Öztoprak et al. 2017)	38 HC 70 ADHD	1000 Hz	Power spectral features	SVM	100

## Methods

**Fig. 1:**
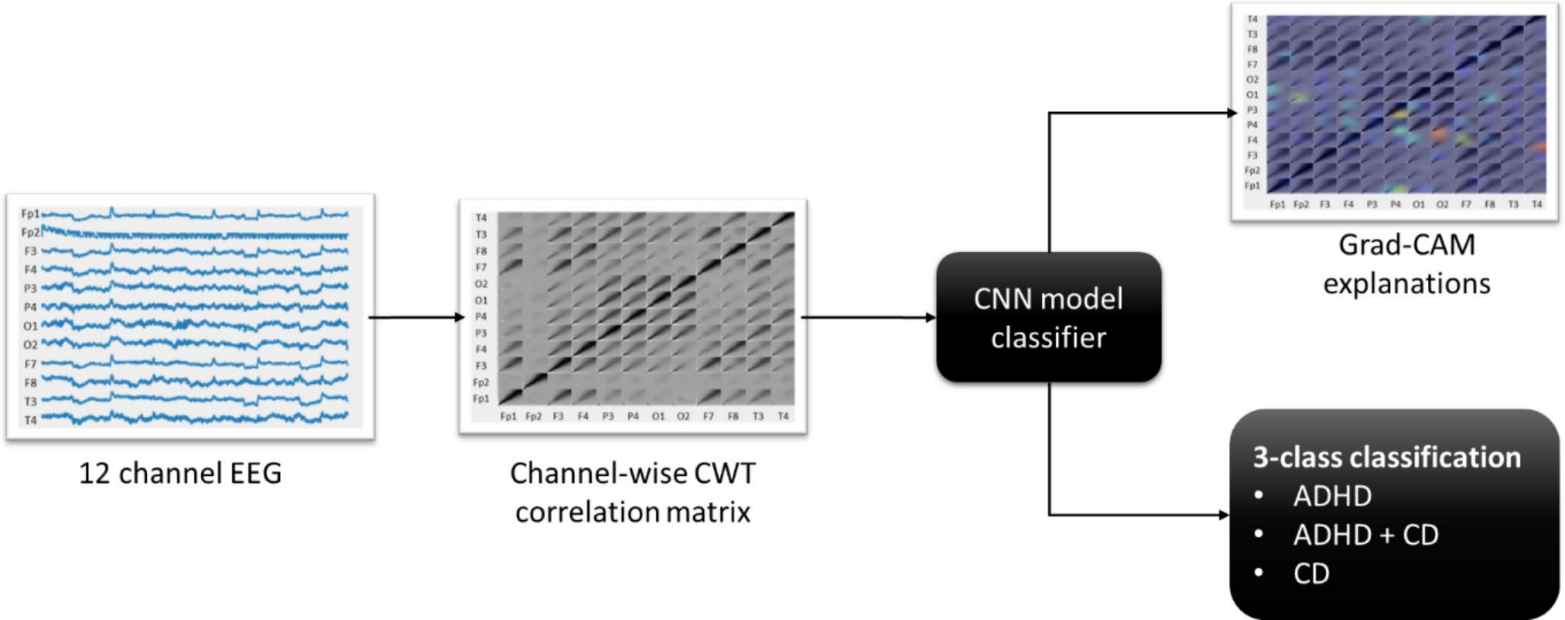
Flowchart process of ADHD/CD-NET

The deep learning system, ADHD/CD-NET, proposed in this study is depicted in Fig. 1. The subsequent sections contain information on the dataset used in this study, describing how we convert 12-channel EEG signals into a channel-wise CWT correlation matrix, expands on the model architecture of ADHD/CD-NET, and introduces Grad-CAM, which is employed to explain ADHD/CD-NET.

### Data acquisition

#### Private dataset (internal evaluation)

The private EEG data for this study came from a clinical trial that was approved by the Domain Specific Review Board (DSRB) of the National Healthcare Group (NHG) in Singapore (DSRB 2008/00410) (Raine et al. 2019). The goal of the trial, which involved 123 participants (7–16 years old) from the Child Guidance Clinic in Singapore, was to determine whether omega-3 supplements have a reduction effect in lowering aggression and whether aggression can be further reduced when the supplements are used in conjunction with standard therapies. In addition to analyzing the efficacy of omega supplements, the trial also obtained ECG data from its participants at the baseline time point. The EEG data were anonymized and de-identified to ensure patient confidentiality. These participants were divided into three groups: CD only (16 children), ADHD only (45 participants), and ADHD + CD (62 participants), following the diagnostic criteria from the Diagnostic and Statistical Manual of Mental Disorders fourth edition Text Revision (DSM-IV-TR). Furthermore, the parents of these children completed a computerized Diagnostic Interview Schedule for Children (DISC), a standard diagnostic test that is widely used in ADHD research and assessment (Lewin et al. 2014). Participants remained resting with their eyes open for 3 min to collect resting-state EEG using MP150 single-channel EEG 100c biopotential amplifiers linked to data acquisition software Acknowledge. The 12 EEG channels aken are as follows: Fp1, Fp2, F3, F4, P3, P4, O1, O2, F7, F8, T3, and T4. As a result, 123 participants’ 12-channel EEG signals with a sampling frequency of 500 Hz were collected, and all 12 channels will be used in this study to develop a multi-channel CAD tool. Each signal is then segmented into 8 chunks of 21.25s epochs, each with 10,625 timesteps ($21.25s\times 500?Hz$), resulting in 128 CD samples ($16 children\times 8 chunks$), 360 ADHD samples($45 children\times 8 chunks$), and 496 ADHD + CD samples ($62 children\times 8 chunks$). The choice of a 21.25-second epoch duration was determined through extensive experimentation involving various segment durations. After thorough testing, it became evident that utilizing a 21.25-second epoch consistently produced the most optimal and favorable results.

#### Public dataset (external evaluation)

This study’s available EEG data comes from (Ali Motie Nasrabadi, Armin Allahverdy, Mehdi Samavati, 2020), which includes 61 children with ADHD and 60 healthy controls (HC), all within the age range of 7 to 12 years old. A qualified psychiatrist diagnosed the ADHD adolescents using DSM-IV criteria, and they were given Ritalin for up to 6 months. There were no psychiatric illnesses, epilepsy, or reports of high-risk behaviors among the children in the HC group. The EEG recording was based on a visual attention test in which the children were given a collection of images with cartoon characters to count. Images were presented instantly and uninterrupted after the youngster provided their response to ensure continuous stimulation during EEG recording. The EEG data were collected at the Psychology and Psychiatry Research Center at Roozbeh Hospital (Tehran, Iran) using 19 channels (Fz, Cz, Pz, C3, T3, C4, T4, Fp1, Fp2, F3, F4, F7, F8, P3, P4, T5, T6, O1, O2) at a sampling frequency of 128 Hz. Similarly, we divided each EEG signal into 16 chunks of 4s epochs, each with 512 timesteps ($4s\times 128?Hz$), yielding 976 ADHD ($61 children\times 16 chunks$) and 960 HC samples ($61 children\times 16 chunks$). Likewise, the utilization of a 4-second epoch duration was determined as optimal through thorough experimentation with various segment durations, consistently showcasing superior results.

### Preprocessing

This subsection explains how EEG signals are converted to scalograms using the continuous wavelet transform (CWT) and the correlations calculated between each channel scalogram, resulting in a channel-wise CWT correlation matrix.

### Continuous wavelets transform (CWT)

Wavelets are a multi-resolution approach that allows for time and frequency fidelities in different frequency bands, making them extremely useful in signal decomposition (Brunton and Kutz 2019). Wavelet fundamentals begin with the mother wavelet $\psi \left(t\right)$, which is described in Eq. 1, where $a$ and $b$ plays the role of scaling and translating the mother wavelet $\psi$, respectively (Brunton and Kutz 2019).

1 $${\psi }_{a,b}\left(t\right)=\frac{1}{\sqrt{a}}\psi \left(\frac{t-b}{a}\right)$$

In CWT, wavelets of varying scales and times are used to shift across the input signal, yielding coefficients that are a function of wavelet scales and shift parameters (Raghavendra et al. 2021). Equation 2, in which the input signal is denoted by $f\left(t\right)$, also describes this CWT mechanism. The resulting transformed signal will be converted into a coefficient matrix of $n \times m$ where $n$ is the total number of scale and $m$ is the length of the signal (Raghavendra et al. 2021). In our study, we have set $n$ = 30 and $m$ = 10,625 which is the timestep of the segmented EEG signal.

2 $${\mathcal{W}}_{\psi }\left(f\right)\left(a,b\right)= ?f,{\psi }_{a,b}?= {\int }_{-\infty }^{\infty }f\left(t\right){\stackrel{-}{\psi }}_{a,b}\left(t\right)dt$$

#### Channel-wise CWT correlation matrix

**Fig. 2:**
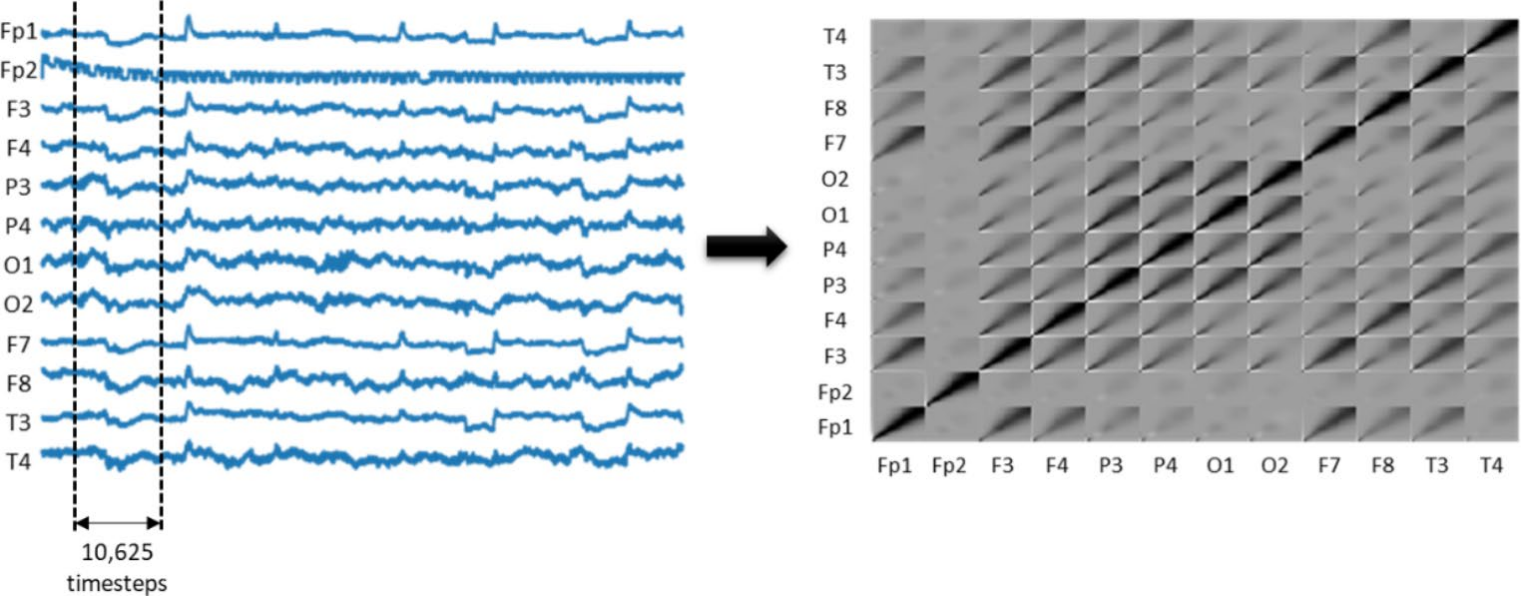
12-Channel EEG segment conversion to channel-wise CWT correlation matrix

PyWavelets (Lee et al. 2019), an open-source wavelet transformation program for Python, was used to apply CWT to each EEG segment, as indicated in Fig. 2. After experimenting with all of the wavelets available in PyWavelets, we chose Gaussian wavelet ‘gaus6’ for our signal transformation as it produced the best results. As a result, for each EEG channel, we were able to obtain a scalogram of size 30 $\times$ 10,625, giving us 360 $\times$ 10,625 for 12 channels. Then, we computed the Pearson correlation coefficient of all the channels, resulting in a channel-wise CWT correlation matrix of size 360 $\times$ 360, which we will use to train our deep learning model.

#### Deep CNN model

**Fig. 3:**
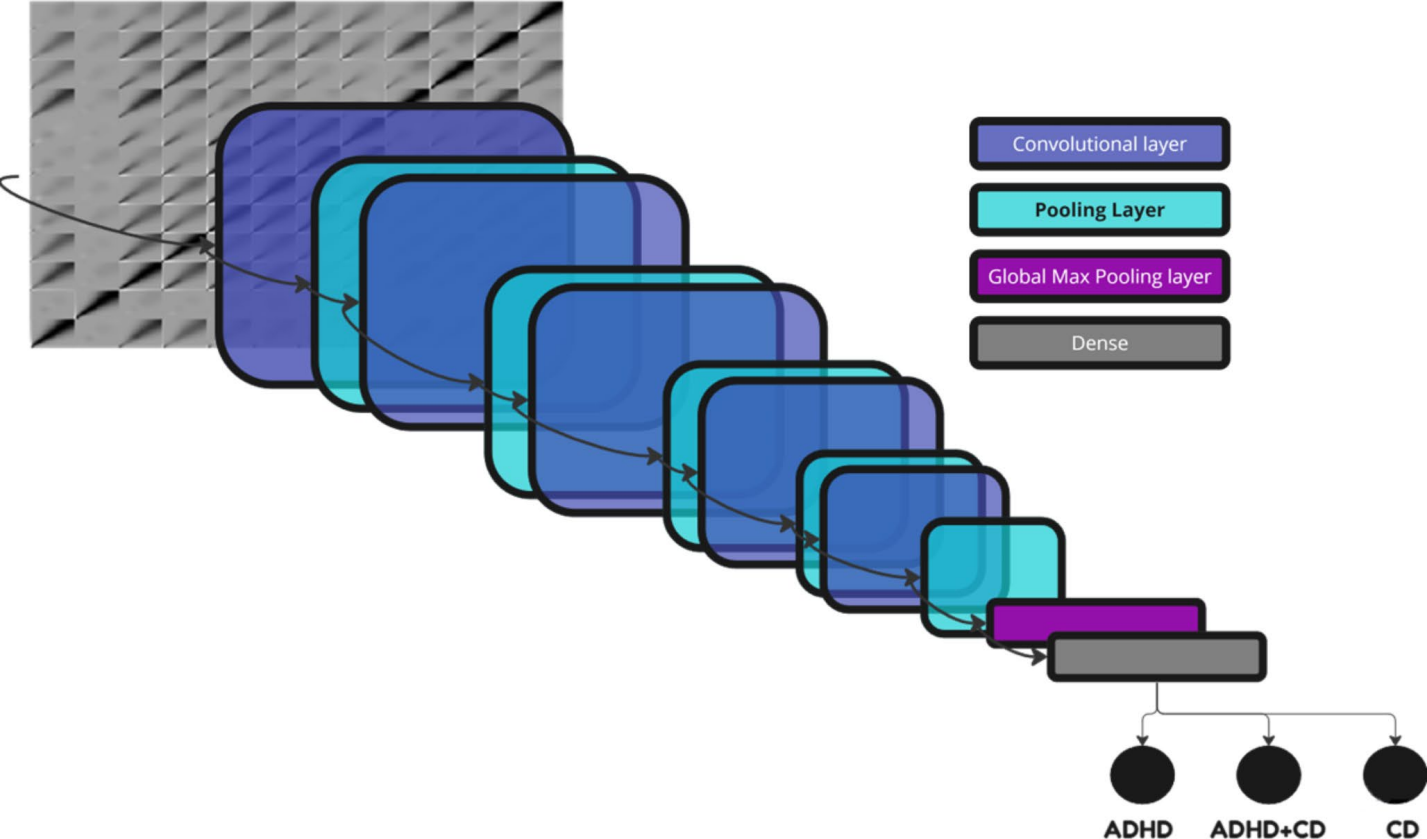
Model architecture of ADHD/CD-NET

After undergoing multiple rounds of hyperparameter tuning, we developed ADHD/CD-NET, illustrated in Fig. 3 and outlined in Table 2. This deep CNN model was specifically designed to classify channel-wise Continuous Wavelet Transform (CWT) correlation matrices into three distinct classes: ADHD, ADHD + CD, and CD. It has been shown in numerous studies that CNN models are suitable in various medical fields that require medical image analysis from CT, MRI, PET and X-ray (Soffer et al. 2019; Yamashita et al. 2018). This is because CNN models are designed to emulate the image recognition abilities of the human visual system (Balderas Silva et al. 2018). In addition to the analysis of medical images, CNN models can also be utilized for biosignals. In this case, there are two approaches: (a) use 1-dimensional (1D) CNN for the full signal analysis or (b) convert the biosignals into 2-dimensional (2D) representation and use 2D-CNN. The latter method was used in this study, where 12-channel EEG data segments were transformed into 2D channel-wise CWT correlation matrices for our proposed 2D CNN model.

**Table 2:** Model layer parameters of ADHD/CD-NET

No.	Layer	Filter no.	Kernel size	Unit	Output
1	Conv2D	8	3 × 3		360 × 360 × 8
2	MaxPooling2D	8		2 × 2	180 × 180 × 8
3	Conv2D	16	3 × 3		180 × 180 × 16
4	MaxPooling2D	16		2 × 2	90 × 90 × 16
5	Conv2D	32	3 × 3		90 × 90 × 32
6	MaxPooling2D	32		2 × 2	45 × 45 × 32
7	Conv2D	64	3 × 3		45 × 45 × 64
8	MaxPooling2D	64		2 × 2	22 × 22 × 64
9	Conv2D	128	3 × 3		22 × 22 × 128
10	MaxPooling2D	64		2 × 2	11 × 11 × 128
11	GlobalMaxPooling2D	128			128
12	Dropout (0.2)				128
13	Dense			32	32
14	BatchNormalization				32
15	Dense (Softmax)			3	3

The convolutional layer, pooling layer, and fully-connected layer were the three key layers that made up a fundamental CNN model. Equation 3 illustrates the operation of the convolutional layer, which converts the input image into a much more simplistic representation for image classification. $S$ represents the input image, $*$ is known as the discrete convolutional operation, and the convolutional kernel’s weight is $W$ which is updated continuously as the kernel moves over the input feature (Albawi et al. 2017; Yildirim et al. 2019). The result of the convolutional layer is the feature map ($O$), which is represented by Eq. 4 where $i$ and $j$ are the feature map’s dimensions (Albawi et al. 2017; Yildirim et al. 2019). The feature map is further simplified by the max pooling layer, which is applied after each convolutional operation. This lowers the feature map’s complexity, lowering the likelihood that the model would overfit (Hafemann et al. 2017). In addition, at layer no. 11 (Fig. 3; Table 2), we have included a global max pooling layer that covers the entire feature map rather than a restricted kernel as in the pooling layer. Hence, the global max pooling layer can significantly reduce the complexity of the feature map compared to a simple max pooling layer.

3 $$\left(S*W\right)\left(i,j\right)=\sum _{m}\sum _{n}S\left(m,n\right)W(i-m,j-n)$$

4 $${O}_{n}^{l}={\left({S}_{W\left(i,j\right)}*W\left(i,j\right)\right)}_{n}$$

In ADHD/CD-NET, the convolution and max pooling operations reduced the input matrix from 360 $\times$ 360 to 11 $\times$ 11, which is then reduced to a 1D array of length 128 after the global max pooling layer. The fully-connected layer, which is the neural network component of the CNN model, will receive this 1D array as input. The fully-connected layer is composed of two layers; the first layer is made up of 32 neurons, while the final output layer is made up of 3 neurons that use the SoftMax activation function to determine the likelihood that the sample would fall into one of three categories: CD, ADHD + CD, or CD. In addition, we have a dropout layer of 0.2, just before the fully connected layer, to lessen the likelihood of model overfitting. As for the optimizer and loss function of the deep CNN model, we used Adam with a learning rate of 0.0001 and sparse categorical cross entropy, respectively. Then we train the model using 700 epochs with a batch size of 15. To address the imbalance in the dataset caused by CD samples being significantly smaller than ADHD and ADHD + CD samples, the weighted loss was also incorporated during model training. This guarantees that the minority CD class is given more weight than the ADHD and ADHD + CD classes, allowing the model to emphasize learning the CD class rather than being overwhelmed by the potential bias created by the big ADHD + CD class. ADHD/CD-NET was created with Python using Tensorflow (v2.9.1). The specifications of the computer used to train the model are Intel Core i9-12900 F CPU, Nvidia QuadroA2000 12GB, 128GB RAM, and 1.0 TB 2.5 Inch SATA SSD.

### Gradient-weighted class activation mapping (Grad-CAM)

Grad-CAM is a well-known XAI method for interpreting CNN models by displaying to users what the CNN model “sees” as a significant characteristic when generating a prediction (Jahmunah et al. 2022; Selvaraju et al. 2016). The distinctive features will gradually become more noticeable as the CNN layers’ convolution operation continue to convolve the feature map; as a result, the last convolutional layer is considered to have the most distinctive feature highlighted. Therefore, Grad-CAM is frequently applied to the final convolution layer to determine which features have been highlighted by utilizing the gradient information provided by the neurons in the convolutional layer as they assign importance value to the region of interest on the feature map (Jahmunah et al. 2022; Selvaraju et al. 2016). Then, Grad-CAM produces a heatmap with the important regions highlighted in red and the less important regions remaining in blue.

## Results

### Internal evaluation results (private dataset)

**Fig. 4:**
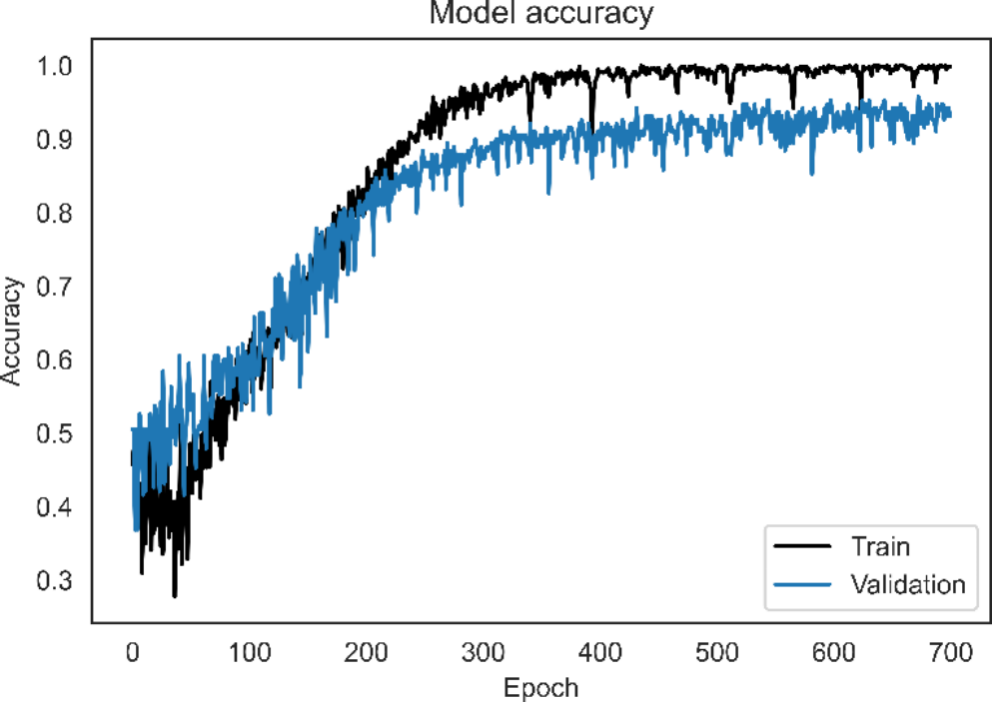
Performance graph of ADHD/CD-NET during model training

We used 10-fold cross-validation to evaluate the model. Figure 4 depicts the model performance graph during training, which demonstrated that the model did not overfit, as seen by the consistently small difference between the training and validation curves. In addition, we used a model checkpoint during model training to save the best-performing model weights obtained during training. The best-performing model weights will then be used to evaluate the test fold in 10-fold cross-validation.

**Fig. 5:**
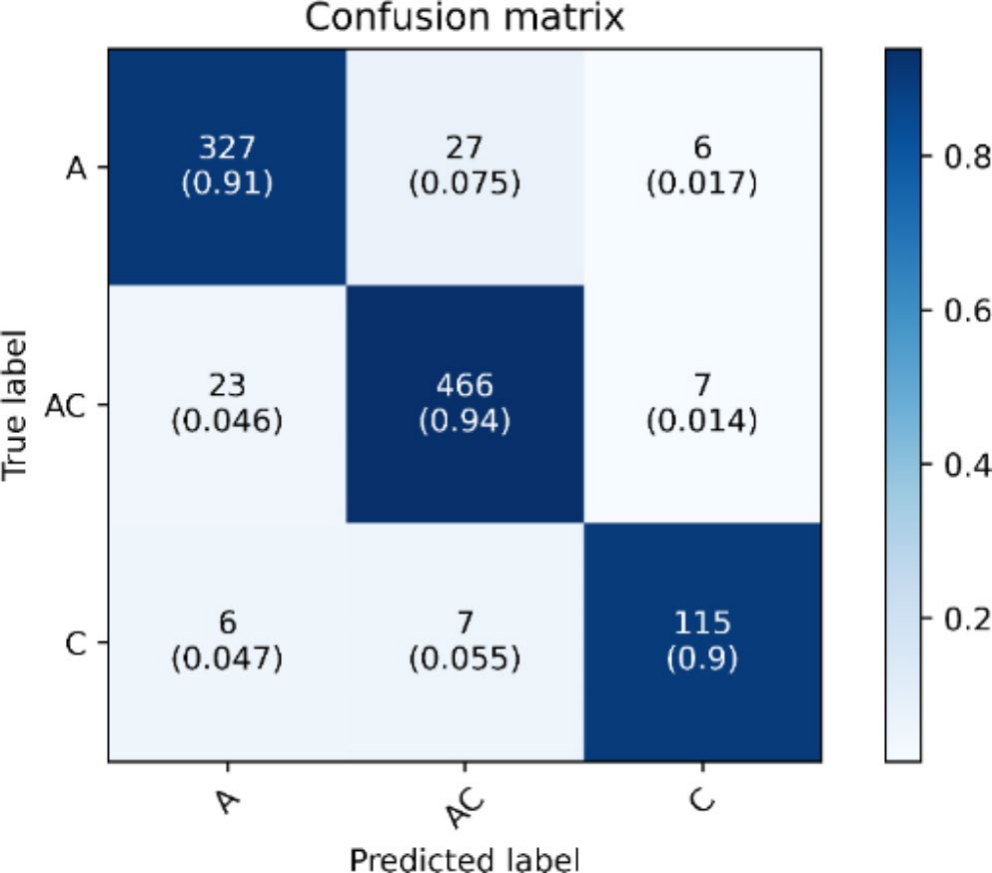
Normalized confusion matrix of ADHD/CD-NET for internal evaluation with our private dataset. ‘A’ represents ADHD samples, ‘AC’ represents ADHD + CD samples, and ‘C’ represents CD samples

The dataset was divided into ten equal folds for 10-fold cross-validation, with 9 folds used for model training and the remaining fold used to evaluate model performance. In addition, we used 1 fold from the training dataset for model tuning as the validation set. This process is repeated ten times to ensure that every fold has gone through model training, validation, and testing. The results of 10-fold cross-validation are shown in Table 3. ADHD/CD-NET achieved a high overall performance of 93.70% classification accuracy. The model also has a high specificity of 95.35%, which means it correctly recognizes the majority of true negative samples rather than misclassifying them as false positives. The remaining metrics are sensitivity and precision. Sensitivity measures how many samples were correctly classified as true positive rather than a false negative, while precision compares the true positive class to the false positive class. Hence, there is always a trade-off between model sensitivity and precision, as high model sensitivity may indicate that the model is able to correctly recognize most of the true positive samples, whereas low model precision indicates that the model incorrectly classifies the majority of other samples as the true positive class. Fortunately, ADHD/CD-NET achieves 90.83% and 91.85% for model sensitivity and precision, indicating that our model can effectively balance the trade-off between sensitivity and precision. The model performance can also be visualized using a confusion matrix, as shown in Fig. 5, which demonstrates that the majority of the samples in each class have been correctly identified even when there is a class imbalance, with the CD class having the fewest samples, but the model could correctly predict 115 out of 128 CD samples.

**Table 3:** Performance parameter of ADHD/CD-NET for internal evaluation with our private dataset

Fold	Accuracy (%)	Sensitivity (%)	Specificity (%)	Precision (%)
1	93.94	91.67	95.24	91.67
2	94.95	94.44	95.24	91.89
3	92.93	86.11	96.83	93.94
4	96.97	97.22	96.82	94.59
5	96.97	94.44	98.41	97.14
6	90.90	94.44	88.89	82.93
7	93.88	91.67	95.16	91.67
8	88.78	75.00	96.77	93.10
9	91.75	91.67	91.80	86.84
10	95.88	91.67	98.36	97.06
Mean ± (SD)	**93.70 ± 2.53**	**90.83 ± 5.96**	**95.35 ± 2.81**	**91.85 ± 4.16**

### Explanation with Grad-CAM (private dataset)

**Fig. 6:**
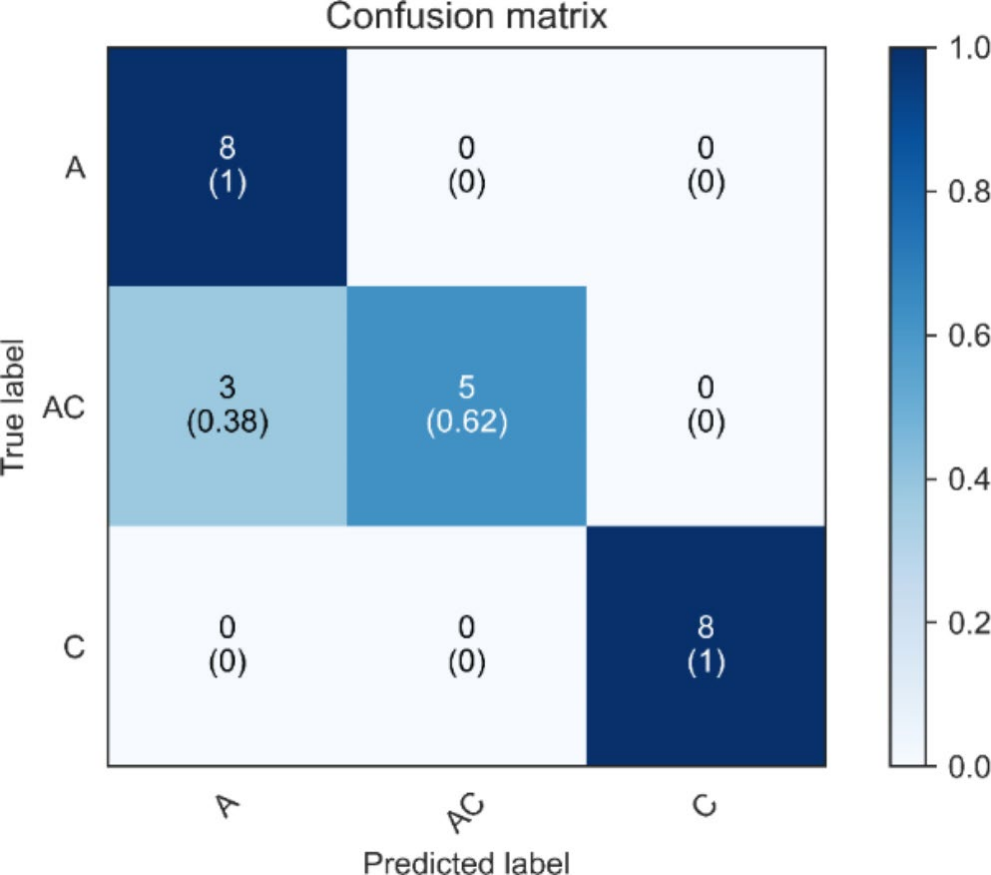
Confusion matrix of ADHD/CD-NET on test set of 3 subjects from each disorder category. ‘A’ represents ADHD samples, ‘AC’ represents ADHD + CD samples, and ‘C’ represents CD samples

**Fig. 7:**
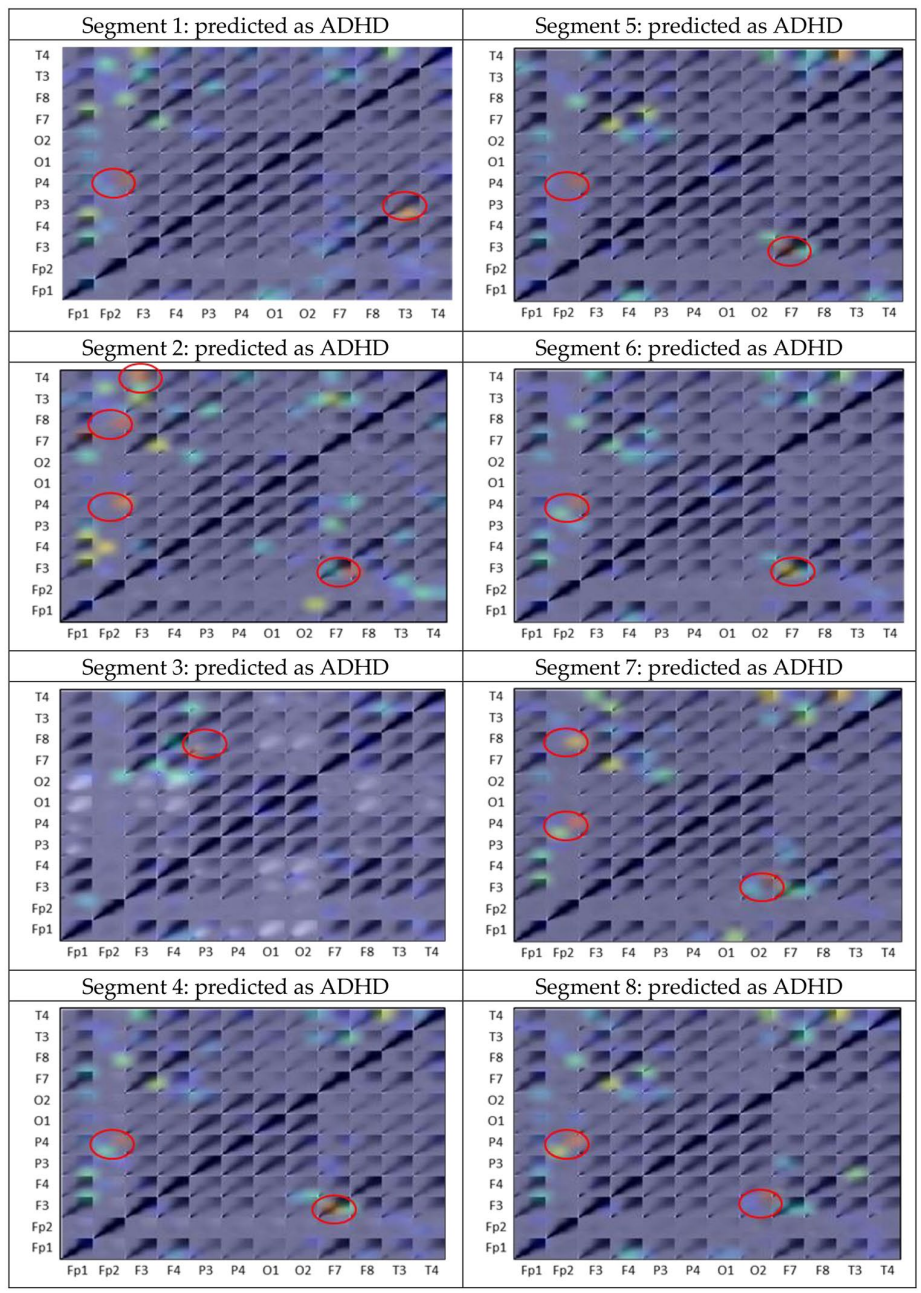
Grad-CAM heatmap produced for each segment’s channel-wise CWT matrix in the ADHD subject. Red circles indicate the EEG channels recognized by ADHD/CD-NET as important for prediction

**Fig. 8:**
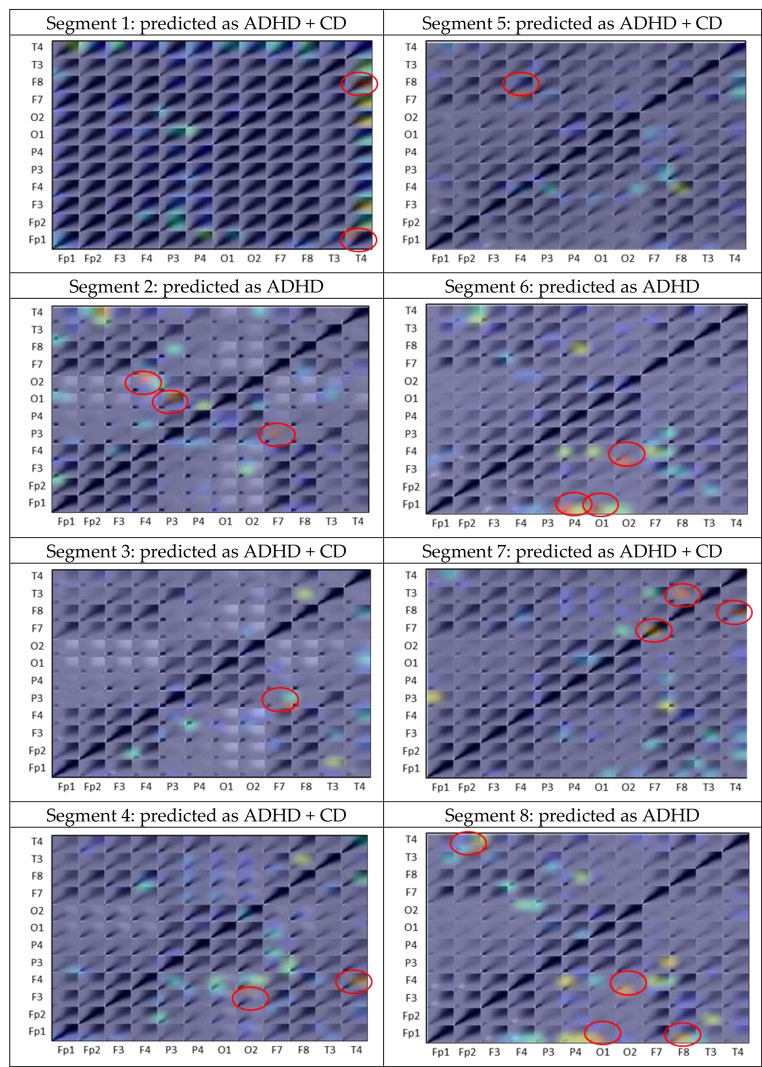
Grad-CAM heatmap produced for each segment’s channel-wise CWT matrix in the ADHD+CD subject. Red circles indicate the EEG channels recognized by ADHD/CD-NET as important for prediction

**Fig. 9:**
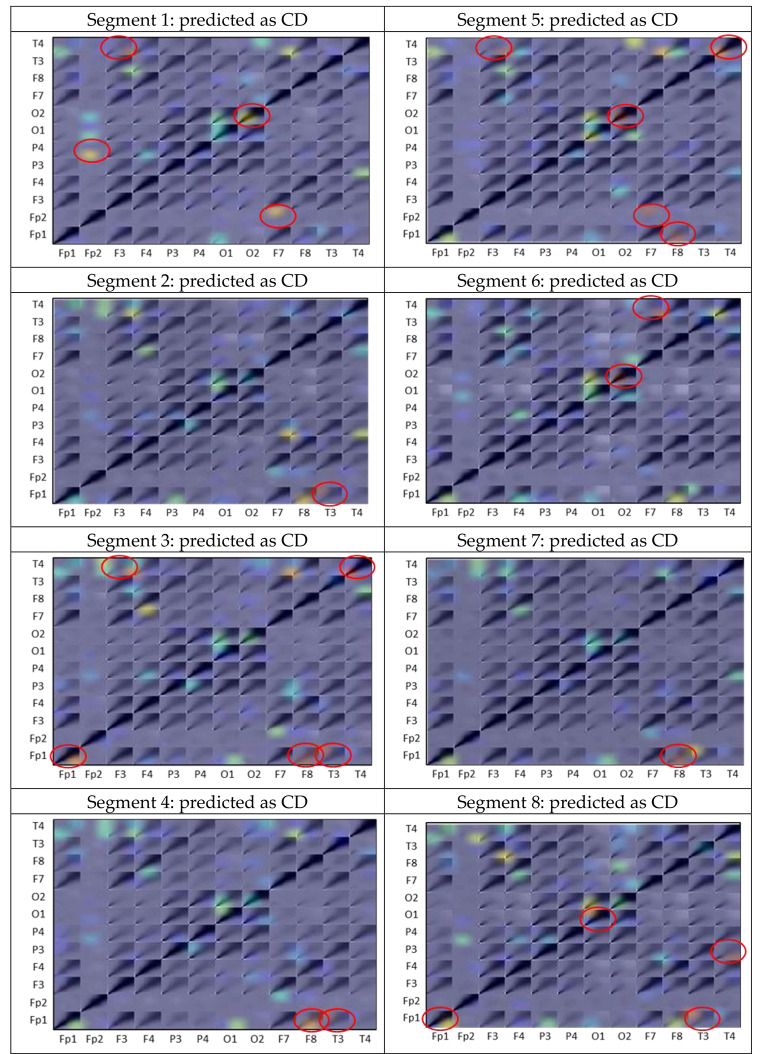
Grad-CAM heatmap produced for each segment’s channel-wise CWT matrix in the CD subject. Red circles indicate the EEG channels recognized by ADHD/CD-NET as important for prediction

To demonstrate the explainability of ADHD/CD-NET, we performed a separate experiment in which we randomly selected one subject from each category of ADHD, ADHD + CD, and CD as the test set, with the remaining subjects used to train the model. This ensures that the test set is entirely new to the model. Recall that we segmented the EEG data of each patient into 8 chunks in Sect. 2.1. Figure 6 depicts the classification result of the model on the test set. As can be seen, the model correctly identifies the ADHD and CD classes with 100% accuracy. The ADHD + CD samples, on the other hand, had three segments misclassified as ADHD. We then applied Grad-CAM to all of the segments and interestingly, we discovered that the correlation between channels Fp2 and P4 appears to be the consistent significant contributors as to why the segment is predicted as ADHD (Fig. 7). Repeating patterns are less visible in ADHD + CD and CD samples, especially in the former, because it contains the combination of ADHD and CD characteristics (Fig. 8). Nonetheless, there are some regions in the CD samples that appear to be significant for predicting CD: the correlation between Fp1 with F8 and Fp1 with T3 (Fig. 9).

### External evaluation results (public dataset)

**Fig. 10:**
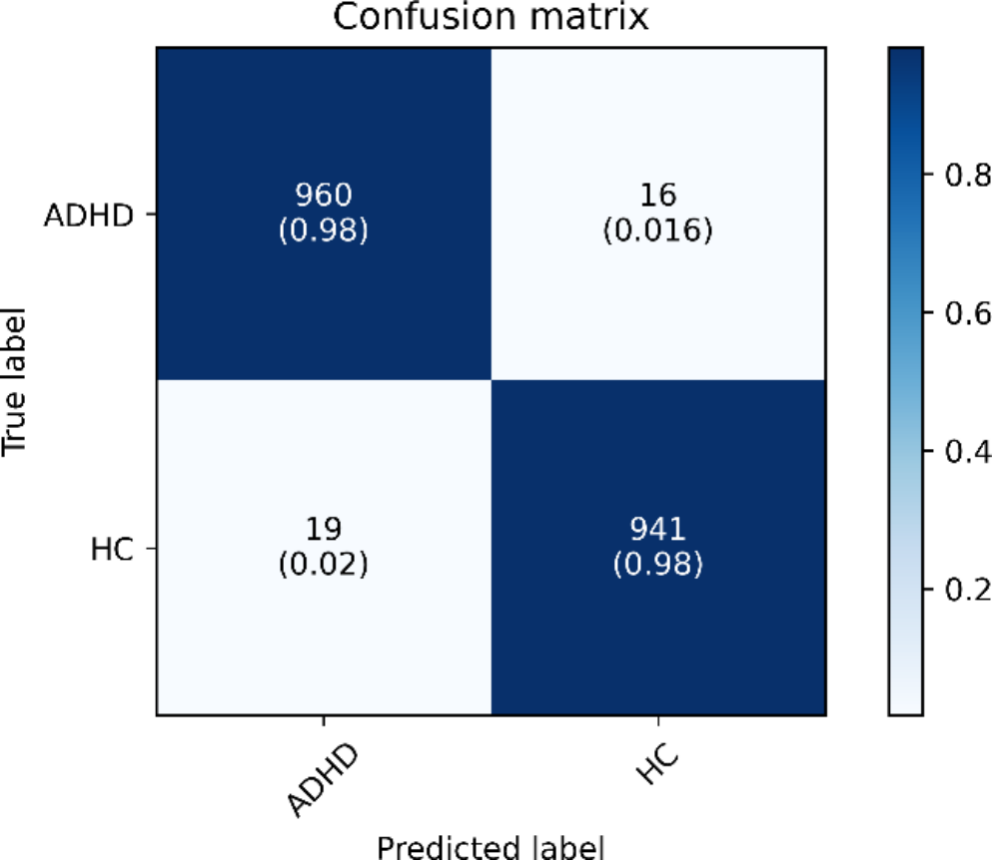
Normalized confusion matrix of ADHD/CD-NET for external evaluation with public dataset

Prior to using an external public dataset to assess ADHD/CD-NET, the weights from earlier training with internal private dataset have already been cleared. This ensures that the ADHD/CD-NET is evaluated with a clean slate and that memory from earlier training does not interfere with its evaluation with an external public dataset. Table 4 displays the findings of 10-fold cross validation for external evaluation. ADHD/CD-NET has a high classification accuracy of 98.19%, sensitivity of 98.36%, specificity of 98.02%, and precision of 98.06%. This demonstrates that ADHD/CD-NET can also perform well with an external dataset and accurately distinguish between ADHD and HC control, as seen in the confusion matrix in Fig. 10; ADHD/CD-NET properly detected 960 out of 976 ADHD samples and 941 out of 960 HC samples.

**Table 4:** Performance parameter of ADHD/CD-NET for external evaluation with public dataset

Fold	Accuracy (%)	Sensitivity (%)	Specificity (%)	Precision (%)
1	97.93	100	95.83	96.08
2	98.97	97.96	100	100
3	98.45	98.98	97.92	97.98
4	98.97	100	97.92	98
5	98.97	98.98	98.96	98.98
6	96.39	95.92	96.88	96.91
7	99.48	100	98.95	98.98
8	98.45	98.96	97.92	97.96
9	97.41	95.88	98.96	98.94
10	96.89	96.91	96.88	96.91
Mean ± (SD)	**98.19 ± 0.96**	**98.36 ± 1.54**	**98.02 ± 1.18**	**98.06 ± 1.13**

## Discussion

This study objectively distinguishes ADHD from CD using EEG signals via our proposed deep learning system, ADHD/CD-NET. The treatment protocol for this three combination of disorders differ; while stimulant and non-stimulant medications are available for ADHD (Brown et al. 2018), there are currently no official U.S. Food and Drug Administration (FDA) medications approved to treat CD (Lillig 2018a). Misdiagnosis of CD as ADHD increases the likelihood of incorrect medication prescription and may also result in a higher risk of drug abuse, as was mentioned earlier, where students may fake ADHD symptoms to obtain stimulant medication (Sansone and Sansone 2011). Therefore, objective ADHD, ADHD + CD, and CD diagnosis is required not only for the benefit of early detection and treatment but also to reduce the likelihood of drug abuse.

Previously, our team proposed ML models using the same dataset as in Table 5. This study is the first to use a DL model with the unique dataset to differentiate ADHD, ADHD + CD, and CD. In (Tor et al. 2021) study, they used empirical mode decomposition and discrete wavelet transform to decompose the signal, then extracted nonlinear features such as entropy, fractal dimension, Lempel-Ziv complexity, and so on. The top few significant features were then selected using a sequential forward selection technique to train their k-nearest neighbour (kNN) ML classifier, resulting in a high classification accuracy of 97.88%. Despite the high model performance, the proposed technique suffers from poor interpretability because nonlinear features of EEG signals are not recognized as a clinical standard for ADHD or CD diagnosis. When they decomposed the EEG signals to extract nonlinear features, information such as time and location of the EEG characteristic contributing to the diagnosis were lost.

**Table 5:** List of studies that used the same private dataset for classification of ADHD, ADHD + CD, and CD

Study	Dataset	Features	Classifier	Accuracy (%)
(Tor et al. 2021)	45 ADHD 62 ADHD + CD 16 CD 500 Hz	EEG (Nonlinear features)	ML (kNN)	97.88
(Koh et al. 2022)		ECG (EWT entropies)	ML (Bagged tree)	87.19
This work		EEG (CWT + correlation matrix)	DL (CNN + Grad-CAM)	93.70

Similarly, (Koh et al. 2022) followed the same procedure to decompose the ECG signals using empirical wavelet transform (EWT) and extracted EWT entropies feature to train their best performing ML classifier, bagged tree, resulting in 87.19% classification accuracy. Likewise, these EWT entropies are not clinically recognized as well. Therefore, this research aims to improve on previous works by incorporating time localization and channel selection. Segmenting EEG signals into chunks of 0-21.25 s, 21.26-42.50 s, 42.51-63.75 s, and so on reveals which EEG segment in time exhibits ADHD or CD characteristics. Grad-CAM’s heatmap provided information on which EEG channel displayed such characteristics. This is due to the fact that EEG signals are highly varying, which means that not all EEG channels may capture the important characteristic contributing to the diagnosis. Similarly, it is possible that not all time segments consistently exhibit the characteristics of ADHD or CD patients. Thus, our study addresses this limitation by identifying the contributing EEG segment and significant channels for ADHD, ADHD + CD, and CD detection, thereby providing objective data for relevant medical professionals in this field, such as psychologists, pediatricians, and neurologists.

Furthermore, we externally evaluated our proposed model with a public dataset (Ali Motie Nasrabadi, Armin Allahverdy, Mehdi Samavati, 2020) to demonstrate that ADHD/CD-NET can discriminate ADHD from HC in addition to ADHD, ADHD + CD, and CD. As a result, ADHD/CD-NET produced equivalent results to earlier research that employed the same public dataset; as shown in Table 6, all studies achieved classification accuracy of greater than 90% using DL models. (Khare and Acharya 2023) achieved the highest classification accuracy of 99.81%, followed by (Talebi and Motie Nasrabadi 2022) with classification accuracy of 99.09%. ADHD/CD-NET, on the other hand, achieved a high comparable classification accuracy of 98.19%. This shows that ADHD/CD-NET can perform in both three-class (ADHD, ADHD + CD, and CD) and binary (ADHD and HC) classification tasks. It can be noted from Table 6 that, the performance of our proposed model is comparable with the state-of-the-art techniques. We have also shown that our model is able to classify ADHD from HC without changing layer parameters using an external public dataset. This justifies that our generated mode is accurate and robust.

**Table 6:** List of studies that used the same public dataset for classification of ADHD and HC

Study	Features	Classifier	Accuracy (%)
(Mohammadi et al. 2016)	EEG (Nonlinear features)	DL (MLP)	93.65
(Allahverdy et al. 2016)	EEG (Nonlinear features)	DL (MLP)	96.70
(Talebi and Motie Nasrabadi 2022)	Linear and nonlinear connectivity features	DL (ANN)	99.09
(Ahire et al. 2023)	DWT and statistical features	ML (Bernoulli Naive Bayes)	96.00
(TaghiBeyglou et al. 2022)	Raw EEG signals	DL (CNN)	95.83
(Atila et al. 2023)	Features extracted based on Sophie Germain’s Primes on Ulam’s Spiral	ML (SVM)	97.46
(Maniruzzaman et al. 2023)	Time domain and morphological features	ML (Gaussian process classification)	97.53
(Khare and Acharya 2023)	VMD-HT extracted EEG features	ML (EBM)	99.81
**This work**	EEG (CWT + correlation matrix)	DL (CNN)	98.19

In summary, the novelties and the significant aspects of our research are listed as follows:

- We are the first study to use an explainable DL model to distinguish between ADHD, ADHD + CD, and CD.
- Another innovative concept is the preprocessing method for transforming EEG into a channel-wise CWT correlation matrix.
- We achieved 93.70% classification accuracy, demonstrating the efficacy of ADHD/CD-NET.
- Grad-CAM was also used to highlight the EEG channels that significantly influenced the classification outcome.
- As a result, ADHD/CD-NET can locate the EEG channels that exhibit abnormal EEG features and provide time localization of such traits.
- ADHD/CD-NET also achieves a high classification accuracy of 98.19% with an external public dataset, separating ADHD samples from HC samples.

Despite the benefits of ADHD/CD-NET, we are nevertheless constrained by issues like the lack of data which compromised the performance of ADHD/CD-NET. The data’s diversity is also another limitation because Singapore is only home to three major races: Chinese, Malays, and Indians. As a result, the data used in this study can only represent the Singaporean population and cannot be generalized (Chong 2007).

Having an objective CAD tool for ADHD and CD diagnosis and differentiation, on the other hand, can significantly reduce the healthcare burden in Singapore’s mental health institutions, where there is a shortage of healthcare professionals; the population of psychiatrists to population ratio is an appallingly low 2.6:100,000 (Chong 2007). Therefore, future work must be undertaken in order to eventually incorporate CAD tools in mental healthcare facilities. As a result of the success of this study, we hope to develop a DL model for ADHD, ADHD + CD, and CD differentiation using ECG signals, allowing for an additional parameter for objective diagnosis in addition to EEG. Furthermore, unlike EEG, the ECG signal can be easily captured by smartwatches as compared to the laborious electrodes needed to record EEG signals. ECG signal preprocessing is significantly less complex than EEG’s, which lowers computational complexity. If such a successful DL system is created, patients with ADHD or CD may be able to monitor their conditions using smartphones. This will help confirm the diagnosis of ADHD because mental health professionals and clinicians will have access to the daily ECG data to see if the patient exhibits any characteristics of ADHD or CD. Hence, the path of future ADHD or CD detection and monitoring systems should shift towards widely accessible physiological data, such as ECG (Khare et al. 2023; Loh et al. 2023). As such, future work will necessarily require the collection of ECG data via wearable devices from ADHD, ADHD + CD, CD, and healthy controls to develop this ADHD or CD detection and monitoring system.

## Conclusion

This work proposes a deep learning system (ADHD/CD-NET) for EEG signal-based detection of CD, ADHD + CD, and ADHD. A unique preprocessing method is used in this proposed system to turn segments of 12 EEG channels into channel-wise CWT correlation matrices, which the deep CNN model then analyzes and categorizes into one of three disorder categories. As a result, ADHD/CD-NET successfully achieved a high classification accuracy of 93.70%, proving that our system can distinguish ADHD, ADHD + CD, and CD, which is difficult for mental health professionals and clinicians to do because they share similar clinical symptoms. Additionally, we use Grad-CAM, which can assist us in highlighting the critical EEG channels to consider for diagnosis. Hence, ADHD/CD-NET is capable of performing time localization from the EEG signal segments in time and selection of significant EEG channels for diagnosis, offering objecting analysis for mental health professionals and clinicians to consider when making a diagnosis. Additionally, we tested ADHD/CD-NET using an external public dataset, and the results showed 98.19% classification accuracy when differentiating ADHD from healthy controls. In the future, we intend to evaluate our model using a larger samples dataset with more patient diversity and additional physiological signals, such as the ECG, which can be easily obtained via smartwatches.

### Electronic supplementary material

Below is the link to the electronic supplementary material.

Supplementary Material 1

## Data Availability

The data and code generated and analysed during the current study are not publicly available for legal/ethical reasons but are available from the corresponding author upon reasonable request.

## References

[CR1] Ahire N, Awale RN, Wagh A (2023) Electroencephalogram (EEG) based prediction of attention deficit hyperactivity disorder (ADHD) using machine learning. Appl Neuropsychology: Adult 1–12. 10.1080/23279095.2023.2247702 10.1080/23279095.2023.2247702 37647332

[CR2] Ahmadi A, Kashefi M, Shahrokhi H, Nazari MA (2021) Computer aided diagnosis system using deep convolutional neural networks for ADHD subtypes. Biomed Signal Process Control 63:102227. 10.1016/j.bspc.2020.102227 10.1016/j.bspc.2020.102227

[CR3] Albawi S, Mohammed TA, Al-Zawi S (2017) Understanding of a convolutional neural network. *2017 International Conference on Engineering and Technology (ICET)*, 1–6. 10.1109/ICEngTechnol.2017.8308186

[CR5] Allahverdy A, Khorrami A, Mohammadi MR, Nasrabadi AM (2016) Detecting ADHD children using the attention continuity as nonlinear feature of EEG. Front Biomedical Technol 3(1–2):28–33

[CR6] Altınkaynak M, Dolu N, Güven A, Pektaş F, Özmen S, Demirci E, İzzetoğlu M (2020) Diagnosis of attention deficit hyperactivity disorder with combined time and frequency features. Biocybernetics and Biomedical Engineering 40(3):927–937. 10.1016/j.bbe.2020.04.006 10.1016/j.bbe.2020.04.006

[CR7] American Psychiatric Association (2013) Diagnostic and statistical Manual of Mental disorders. American Psychiatric Association. 10.1176/appi.books.9780890425596

[CR8] Arnsten AFT (2009) ADHD and the Prefrontal Cortex. J Pediatr 154(5). 10.1016/j.jpeds.2009.01.018. I-S43 10.1016/j.jpeds.2009.01.018 PMC2894421 20596295

[CR9] Atila O, Deniz E, Ari A, Sengur A, Chakraborty S, Barua PD, Acharya UR (2023) LSGP-USFNet: automated attention deficit hyperactivity disorder detection using locations of Sophie Germain’s primes on Ulam’s spiral-based features with Electroencephalogram signals. Sensors 23(16):7032. 10.3390/s23167032 37631569 10.3390/s23167032 PMC10459515

[CR10] Balderas Silva D, Ponce Cruz P, Molina Gutierrez A (2018) Are the long–short term memory and convolution neural networks really based on biological systems? ICT Express 4(2):100–106. 10.1016/j.icte.2018.04.001 10.1016/j.icte.2018.04.001

[CR11] Barredo Arrieta A, Díaz-Rodríguez N, Del Ser J, Bennetot A, Tabik S, Barbado A, Garcia S, Gil-Lopez S, Molina D, Benjamins R, Chatila R, Herrera F (2020) Explainable Artificial Intelligence (XAI): concepts, taxonomies, opportunities and challenges toward responsible AI. Inform Fusion 58:82–115. 10.1016/j.inffus.2019.12.012 10.1016/j.inffus.2019.12.012

[CR12] Barua PD, Dogan S, Baygin M, Tuncer T, Palmer EE, Ciaccio EJ, Acharya UR (2022) TMP19: a Novel Ternary Motif Pattern-based ADHD detection model using EEG signals. Diagnostics 12(10):2544. 10.3390/diagnostics12102544 36292233 10.3390/diagnostics12102544 PMC9600696

[CR13] Biederman J, Newcorn J, Sprich S (1991) Comorbidity of attention deficit hyperactivity disorder with conduct, depressive, anxiety, and other disorders. Am J Psychiatry 148(5):564–577. 10.1176/ajp.148.5.564 2018156 10.1176/ajp.148.5.564

[CR14] Brown KA, Samuel S, Patel DR (2018) Pharmacologic management of attention deficit hyperactivity disorder in children and adolescents: a review for practitioners. Translational Pediatr 7(1):36–47. 10.21037/tp.2017.08.02 10.21037/tp.2017.08.02 PMC5803014 29441281

[CR15] Brunton SL, Kutz JN (2019) Data-Driven Science and Engineering. Cambridge University Press. 10.1017/9781108380690

[CR16] Carpentier P-J, Knapen LJM, van Gogh MT, Buitelaar JK, De Jong CAJ (2012) Addiction in Developmental Perspective: influence of Conduct Disorder Severity, Subtype, and attention-deficit hyperactivity disorder on Problem Severity and Comorbidity in adults with opioid dependence. J Addict Dis 31(1):45–59. 10.1080/10550887.2011.642756 22356668 10.1080/10550887.2011.642756

[CR17] Catherine Joy R, George T, Rajan S., Albert, A., Subathra MSP (2022) Detection of ADHD from EEG signals using different Entropy measures and ANN. Clin EEG Neurosci 53(1):12–23. 10.1177/15500594211036788 34424101 10.1177/15500594211036788

[CR19] Chen H, Song Y, Li X (2019a) A deep learning framework for identifying children with ADHD using an EEG-based brain network. Neurocomputing 356:83–96. 10.1016/j.neucom.2019.04.058 10.1016/j.neucom.2019.04.058

[CR20] Chen H, Song Y, Li X (2019b) Use of deep learning to detect personalized spatial-frequency abnormalities in EEGs of children with ADHD. J Neural Eng 16(6):066046. 10.1088/1741-2552/ab3a0a 31398717 10.1088/1741-2552/ab3a0a

[CR18] Chen H, Chen W, Song Y, Sun L, Li X (2019c) EEG characteristics of children with attention-deficit/hyperactivity disorder. Neuroscience 406:444–456. 10.1016/j.neuroscience.2019.03.048 30926547 10.1016/j.neuroscience.2019.03.048

[CR21] Chong S-A (2007) Mental healthcare in Singapore. Int Psychiatry: Bull Board Int Affairs Royal Coll Psychiatrists 4(4):88–90. http://www.ncbi.nlm.nih.gov/pubmed/31507910 10.1192/S1749367600005257 PMC6734783 31507910

[CR22] Dubreuil-Vall L, Ruffini G, Camprodon JA (2020) Deep learning convolutional neural networks discriminate adult ADHD from healthy individuals on the basis of event-related spectral EEG. Front NeuroSci 14. 10.3389/fnins.2020.00251 10.3389/fnins.2020.00251 PMC7160297 32327965

[CR23] FARAONE SV, BIEDERMAN J, JETTON, J. G., TSUANG MT (1997) Attention deficit disorder and conduct disorder: longitudinal evidence for a familial subtype. Psychol Med 27(2):291–300. 10.1017/S0033291796004515 9089822 10.1017/S0033291796004515

[CR24] Faust O, Razaghi H, Barika R, Ciaccio EJ, Acharya UR (2019) A review of automated sleep stage scoring based on physiological signals for the new millennia. Comput Methods Programs Biomed 176:81–91. 10.1016/j.cmpb.2019.04.032 31200914 10.1016/j.cmpb.2019.04.032

[CR25] Guney G, Kisacik E, Kalaycioglu C, Saygili G (2021) Exploring the attention process differentiation of attention deficit hyperactivity disorder (ADHD) symptomatic adults using artificial intelligence on electroencephalography (EEG) signals. TURKISH J Electr Eng Comput Sci 29(5):2312–2325. 10.3906/elk-2011-3 10.3906/elk-2011-3

[CR26] Hafemann LG, Sabourin R, Oliveira LS (2017) Learning features for offline handwritten signature verification using deep convolutional neural networks. Pattern Recogn 70:163–176. 10.1016/j.patcog.2017.05.012 10.1016/j.patcog.2017.05.012

[CR27] Hall KM, Irwin MM, Bowman KA, Frankenberger W, Jewett DC (2005) Illicit use of prescribed stimulant medication among College Students. J Am Coll Health 53(4):167–174. 10.3200/JACH.53.4.167-174 15663065 10.3200/JACH.53.4.167-174

[CR28] Jahmunah V, Ng EYK, Tan R-S, Oh SL, Acharya UR (2022) Explainable detection of Myocardial Infarction using deep learning models with Grad-CAM technique on ECG signals. Comput Biol Med 146:105550. 10.1016/j.compbiomed.2022.105550 35533457 10.1016/j.compbiomed.2022.105550

[CR29] Kaur S, Singh S, Arun P, Kaur D, Bajaj M (2020) Phase Space Reconstruction of EEG signals for classification of ADHD and control adults. Clin EEG Neurosci 51(2):102–113. 10.1177/1550059419876525 31533446 10.1177/1550059419876525

[CR30] Khare SK, Acharya UR (2023) An explainable and interpretable model for attention deficit hyperactivity disorder in children using EEG signals. Comput Biol Med 155:106676. 10.1016/j.compbiomed.2023.106676 36827785 10.1016/j.compbiomed.2023.106676

[CR31] Khare SK, March S, Barua PD, Gadre VM, Acharya UR (2023) Application of data fusion for automated detection of children with developmental and mental disorders: a systematic review of the last decade. Inform Fusion 99:101898. 10.1016/j.inffus.2023.101898 10.1016/j.inffus.2023.101898

[CR32] Khoshnoud S, Nazari MA, Shamsi M (2018) Functional brain dynamic analysis of ADHD and control children using nonlinear dynamical features of EEG signals. J Integr Neurosci 17(1). 10.31083/JIN-170033 10.31083/JIN-170033 29172003

[CR33] Kim S, Lee H-K, Lee K (2021) Can the MMPI predict adult ADHD? An Approach using machine learning methods. Diagnostics 11(6):976. 10.3390/diagnostics11060976 34071385 10.3390/diagnostics11060976 PMC8229212

[CR34] Koh JEW, Ooi CP, Lim-Ashworth NS, Vicnesh J, Tor HT, Lih OS, Tan R-S, Acharya UR, Fung DS S (2022) Automated classification of attention deficit hyperactivity disorder and conduct disorder using entropy features with ECG signals. Comput Biol Med 140:105120. 10.1016/j.compbiomed.2021.105120 34896884 10.1016/j.compbiomed.2021.105120

[CR35] KUHNE M, SCHACHAR, R., TANNOCK R (1997) Impact of Comorbid Oppositional or Conduct problems on attention-deficit hyperactivity disorder. J Am Acad Child Adolesc Psychiatry 36(12):1715–1725. 10.1097/00004583-199712000-00020 9401333 10.1097/00004583-199712000-00020

[CR36] Lee G, Gommers R, Waselewski F, Wohlfahrt K, O’Leary A (2019) PyWavelets: a Python package for wavelet analysis. J Open Source Softw 4(36):1237. 10.21105/joss.01237 10.21105/joss.01237

[CR37] Levy F (2014) DSM-5, ICD-11, RDoC and ADHD diagnosis. Australian & New Zealand Journal of Psychiatry 48(12):1163–1164. 10.1177/0004867414557527 25366330 10.1177/0004867414557527

[CR38] Lewin AB, Mink JW, Bitsko RH, Holbrook JR, Parker-Athill EC, Hanks C, Storch EA, Augustine EF, Adams HR, Vierhile AE, Thatcher AR, Murphy TK (2014) Utility of the diagnostic interview schedule for children for assessing Tourette Syndrome in Children. J Child Adolesc Psychopharmacol 24(5):275–284. 10.1089/cap.2013.0128 24813854 10.1089/cap.2013.0128 PMC4064722

[CR39] Lillig M (2018a) Conduct Disorder: Recognition and Management. Am Family Phys 98(10):584–592. http://www.ncbi.nlm.nih.gov/pubmed/30365289 30365289

[CR40] Lillig M (2018b) Conduct Disorder: Recognition and Management. Am Family Phys 98(10):584–592. http://www.ncbi.nlm.nih.gov/pubmed/30365289 30365289

[CR44] Loh HW, Ooi CP, Vicnesh J, Oh SL, Faust O, Gertych A, Acharya UR (2020) Automated detection of Sleep stages using deep learning techniques: a systematic review of the last decade (2010–2020). Appl Sci 10(24):8963. 10.3390/app10248963 10.3390/app10248963

[CR41] Loh HW, Ooi CP, Barua PD, Palmer EE, Molinari F, Acharya UR (2022a) Automated detection of ADHD: current trends and future perspective. Comput Biol Med 146:105525. 10.1016/j.compbiomed.2022.105525 35468405 10.1016/j.compbiomed.2022.105525

[CR43] Loh HW, Ooi CP, Seoni S, Barua PD, Molinari F, Acharya UR (2022b) Application of explainable artificial intelligence for healthcare: a systematic review of the last decade (2011–2022). Comput Methods Programs Biomed 226:107161. 10.1016/j.cmpb.2022.107161 36228495 10.1016/j.cmpb.2022.107161

[CR42] Loh HW, Ooi CP, Oh SL, Barua PD, Tan YR, Molinari F, March S, Acharya UR, Fung DSS (2023) Deep neural network technique for automated detection of ADHD and CD using ECG signal. Comput Methods Programs Biomed 241:107775. 10.1016/j.cmpb.2023.107775 37651817 10.1016/j.cmpb.2023.107775

[CR45] Loo SK, Barkley RA (2005) Clinical utility of EEG in attention deficit hyperactivity disorder. Appl Neuropsychol 12(2):64–76. 10.1207/s15324826an1202_2 16083395 10.1207/s15324826an1202_2

[CR46] Magnus W, Nazir S, Anilkumar AC, Shaban K (2021) Attention Deficit Hyperactivity Disorder. In *StatPearls*. http://www.ncbi.nlm.nih.gov/pubmed/28722868 28722868

[CR47] Maniruzzaman M, Hasan MAM, Asai N, Shin J (2023) Optimal channels and features selection based ADHD detection from EEG Signal using statistical and machine learning techniques. IEEE Access 11:33570–33583. 10.1109/ACCESS.2023.3264266 10.1109/ACCESS.2023.3264266

[CR48] Marshall P, Hoelzle J, Nikolas M (2021) Diagnosing Attention-Deficit/Hyperactivity disorder (ADHD) in young adults: a qualitative review of the utility of assessment measures and recommendations for improving the diagnostic process. Clin Neuropsychol 35(1):165–198. 10.1080/13854046.2019.1696409 31791193 10.1080/13854046.2019.1696409

[CR49] Mattfeld AT, Gabrieli JDE, Biederman J, Spencer T, Brown A, Kotte A, Kagan E, Whitfield-Gabrieli S (2014) Brain differences between persistent and remitted attention deficit hyperactivity disorder. Brain 137(9):2423–2428. 10.1093/brain/awu137 24916335 10.1093/brain/awu137

[CR50] McDuff DR, Baron D (2005) Substance use in Athletics: a sports Psychiatry Perspective. Clin Sports Med 24(4):885–897. 10.1016/j.csm.2005.06.004 16169452 10.1016/j.csm.2005.06.004

[CR51] Mirza B, Wang W, Wang J, Choi H, Chung NC, Ping P (2019) Machine Learning and Integrative Analysis of Biomedical Big Data. Genes 10(2):87. 10.3390/genes10020087 30696086 10.3390/genes10020087 PMC6410075

[CR52] Moghaddari M, Lighvan MZ, Danishvar S (2020) Diagnose ADHD disorder in children using convolutional neural network based on continuous mental task EEG. Comput Methods Programs Biomed 197:105738. 10.1016/j.cmpb.2020.105738 32927404 10.1016/j.cmpb.2020.105738

[CR53] Mohammadi MR, Khaleghi A, Nasrabadi AM, Rafieivand S, Begol M, Zarafshan H (2016) EEG classification of ADHD and normal children using non-linear features and neural network. Biomed Eng Lett 6(2):66–73. 10.1007/s13534-016-0218-2 10.1007/s13534-016-0218-2

[CR54] Müller A, Vetsch S, Pershin I, Candrian G, Baschera G-M, Kropotov JD, Kasper J, Rehim HA, Eich D (2020) EEG/ERP-based biomarker/neuroalgorithms in adults with ADHD: development, reliability, and application in clinical practice. World J Biol Psychiatry 21(3):172–182. 10.1080/15622975.2019.1605198 30990349 10.1080/15622975.2019.1605198

[CR4] Nasrabadi AM, Allahverdy A, Samavati M, M. R. M (2020) EEG data for ADHD / control children. IEEE Dataport. 10.21227/rzfh-zn36 10.21227/rzfh-zn36

[CR55] Nazar M, Alam MM, Yafi E, Su’ud MM (2021) A systematic review of Human–Computer Interaction and Explainable Artificial Intelligence in Healthcare with Artificial Intelligence techniques. IEEE Access 9:153316–153348. 10.1109/ACCESS.2021.3127881 10.1109/ACCESS.2021.3127881

[CR56] Öztoprak H, Toycan M, Alp YK, Arıkan O, Doğutepe E, Karakaş S (2017) Machine-based classification of ADHD and nonADHD participants using time/frequency features of event-related neuroelectric activity. Clin Neurophysiol 128(12):2400–2410. 10.1016/j.clinph.2017.09.105 29096213 10.1016/j.clinph.2017.09.105

[CR57] Piran N, Robinson SR (2006) Associations between disordered eating behaviors and licit and illicit substance use and abuse in a university sample. Addict Behav 31(10):1761–1775. 10.1016/j.addbeh.2005.12.021 16448780 10.1016/j.addbeh.2005.12.021

[CR58] Rabiner DL, Anastopoulos AD, Costello EJ, Hoyle RH, Swartzwelder HS (2010) Predictors of nonmedical ADHD medication use by College Students. J Atten Disord 13(6):640–648. 10.1177/1087054709334505 19465730 10.1177/1087054709334505 PMC3837450

[CR59] Raghavendra U, Gudigar A, Chakole Y, Kasula P, Subha DP, Kadri NA, Ciaccio EJ, Acharya UR (2021) Automated detection and screening of depression using continuous wavelet transform with electroencephalogram signals. Expert Syst. 10.1111/exsy.12803 10.1111/exsy.12803

[CR60] Raine A, Ang RP, Choy O, Hibbeln JR, Ho RM-H, Lim CG, Lim-Ashworth NSJ, Ling S, Liu JCJ, Ooi YP, Tan YR, Fung DS S (2019) Omega-3 (ω -3) and social skills interventions for reactive aggression and childhood externalizing behavior problems: a randomized, stratified, double-blind, placebo-controlled, factorial trial. Psychol Med 49(2):335–344. 10.1017/S0033291718000983 29743128 10.1017/S0033291718000983

[CR61] Rezaeezadeh M, Shamekhi S, Shamsi M (2020) Attention deficit hyperactivity disorder diagnosis using non-linear univariate and multivariate EEG measurements: a preliminary study. Phys Eng Sci Med 43(2):577–592. 10.1007/s13246-020-00858-3 32524443 10.1007/s13246-020-00858-3

[CR62] Salekin RT (2016) Psychopathy in childhood: toward better informing the DSM–5 and ICD-11 conduct disorder specifiers. Personality Disorders: Theory Research and Treatment 7(2):180–191. 10.1037/per0000150 10.1037/per0000150 26389622

[CR63] Sansone RA, Sansone LA (2011) Faking attention deficit hyperactivity disorder. Innovations in Clinical Neuroscience 8(8):10–13. http://www.ncbi.nlm.nih.gov/pubmed/21922064 21922064 PMC3173757

[CR64] Sayal K, Prasad V, Daley D, Ford T, Coghill D (2018) ADHD in children and young people: prevalence, care pathways, and service provision. The Lancet Psychiatry 5(2):175–186. 10.1016/S2215-0366(17)30167-0 29033005 10.1016/S2215-0366(17)30167-0

[CR65] Selvaraju RR, Cogswell M, Das A, Vedantam R, Parikh D, Batra D (2016) Grad-CAM: visual explanations from deep networks via gradient-based localization. 10.1007/s11263-019-01228-7

[CR66] Shaw M, Hodgkins P, Caci H, Young S, Kahle J, Woods AG, Arnold LE (2012) A systematic review and analysis of long-term outcomes in attention deficit hyperactivity disorder: effects of treatment and non-treatment. BMC Med 10(1):99. 10.1186/1741-7015-10-99 22947230 10.1186/1741-7015-10-99 PMC3520745

[CR67] Soffer S, Ben-Cohen A, Shimon O, Amitai MM, Greenspan H, Klang E (2019) Convolutional neural networks for radiologic images: a radiologist’s guide. Radiology 290(3):590–606. 10.1148/radiol.2018180547 30694159 10.1148/radiol.2018180547

[CR68] Sridhar C, Bhat S, Acharya UR, Adeli H, Bairy GM (2017) Diagnosis of attention deficit hyperactivity disorder using imaging and signal processing techniques. Comput Biol Med 88:93–99. 10.1016/j.compbiomed.2017.07.009 28709145 10.1016/j.compbiomed.2017.07.009

[CR69] TaghiBeyglou B, Shahbazi A, Bagheri F, Akbarian S, Jahed M (2022) Detection of ADHD cases using CNN and classical classifiers of raw EEG. Comput Methods Programs Biomed Update 2:100080. 10.1016/j.cmpbup.2022.100080 10.1016/j.cmpbup.2022.100080

[CR70] Talebi N, Motie Nasrabadi A (2022) Investigating the discrimination of linear and nonlinear effective connectivity patterns of EEG signals in children with Attention-Deficit/Hyperactivity disorder and typically developing children. Comput Biol Med 148:105791. 10.1016/j.compbiomed.2022.105791 35863245 10.1016/j.compbiomed.2022.105791

[CR71] TAYLOR E, CHADWICK O, HEPTINSTALL, E., DANCKAERTS M (1996) Hyperactivity and Conduct problems as risk factors for Adolescent Development. J Am Acad Child Adolesc Psychiatry 35(9):1213–1226. 10.1097/00004583-199609000-00019 8824065 10.1097/00004583-199609000-00019

[CR72] Tor HT, Ooi CP, Lim-Ashworth NS, Wei JKE, Jahmunah V, Oh SL, Acharya UR, Fung DS S (2021) Automated detection of conduct disorder and attention deficit hyperactivity disorder using decomposition and nonlinear techniques with EEG signals. Comput Methods Programs Biomed 200:105941. 10.1016/j.cmpb.2021.105941 33486340 10.1016/j.cmpb.2021.105941

[CR73] Tosun M (2021) Effects of spectral features of EEG signals recorded with different channels and recording statuses on ADHD classification with deep learning. Phys Eng Sci Med 44(3):693–702. 10.1007/s13246-021-01018-x 34043150 10.1007/s13246-021-01018-x

[CR74] Travell C, Visser J (2006) ADHD does bad stuff to you’: young people’s and parents’ experiences and perceptions of attention deficit hyperactivity disorder (ADHD). Emotional and Behavioural Difficulties 11(3):205–216. 10.1080/13632750600833924 10.1080/13632750600833924

[CR75] Vahid A, Bluschke A, Roessner V, Stober S, Beste C (2019) Deep learning based on event-related EEG differentiates children with ADHD from healthy controls. J Clin Med 8(7):1055. 10.3390/jcm8071055 31330961 10.3390/jcm8071055 PMC6679086

[CR76] Valo S, Tannock R (2010) Diagnostic instability of DSM–IV ADHD subtypes: effects of Informant Source, Instrumentation, and methods for combining Symptom reports. J Clin Child Adolesc Psychol 39(6):749–760. 10.1080/15374416.2010.517172 21058123 10.1080/15374416.2010.517172

[CR77] Wang C, Wang X, Jing X, Yokoi H, Huang W, Zhu M, Chen S, Li G (2022) Towards high-accuracy classifying attention-deficit/hyperactivity disorders using CNN-LSTM model. J Neural Eng 19(4):046015. 10.1088/1741-2552/ac7f5d 10.1088/1741-2552/ac7f5d 35797967

[CR78] Yamashita R, Nishio M, Do RKG, Togashi K (2018) Convolutional neural networks: an overview and application in radiology. Insights into Imaging 9(4):611–629. 10.1007/s13244-018-0639-9 29934920 10.1007/s13244-018-0639-9 PMC6108980

[CR79] Yildirim O, Baloglu UB, Acharya UR (2019) A deep learning model for automated sleep stages classification using PSG signals. Int J Environ Res Public Health 16(4). 10.3390/ijerph16040599 10.3390/ijerph16040599 PMC6406978 30791379

[CR80] Zhou B, Khosla A, Lapedriza A, Oliva A, Torralba A (2015) *Learning Deep Features for Discriminative Localization*. http://arxiv.org/abs/1512.04150

[CR81] Zhou D, Liao Z, Chen R (2022) Deep learning enabled diagnosis of children’s ADHD based on the Big data of video screen long-range EEG. J Healthc Eng 2022:1–9. 10.1155/2022/5222136 10.1155/2022/5222136 PMC9001066 35419186

