# Supplementary material for: ADHD/CD-NET: automated EEG-based characterization of ADHD and CD using explainable deep neural network technique
Source: Cogn Neurodyn. 2023 Nov 28;18(4):1609–25. doi: 10.1007/s11571-023-10028-2 (PMC11297883; doi:10.1007/s11571-023-10028-2)
Supplement: Supplementary file 1 — Supplementary Material 1 [file 11571_2023_10028_MOESM1_ESM.docx]

**ADHD/CD-NET: Automated EEG-based characterization of ADHD and CD using explainable deep neural network technique**

**Hui Wen Loh, Chui Ping Ooi, Shu Lih Oh, Prabal Datta Barua, Yi Ren Tan, U. Rajendra Acharya, Daniel Shuen Sheng Fung**

**Journal: Cognitive Neurodynamics**

**Manuscript ID: CODY-D-23-00225R2**

We sincerely thank the Editor and Reviewers for their helpful comments and suggestions for improving our manuscript. In the revised version of the manuscript, we have marked in yellow the changes. We sincerely hope the Editor and Reviewers will positively consider our efforts in improving the overall quality and novelty of our work.

Reviewer's Responses to Questions

**Reviewer #1: The author answered my question. However, in terms of innovation, accuracy, and other aspects, it is recommended to do more work.**

Thank you for the comment. Given the pilot nature of this study, it's important to acknowledge that the classification accuracy may be compromised due to the constraints posed by the relatively small dataset size. Nevertheless, we made diligent efforts to maximize the potential of this dataset by meticulously considering every EEG channel and minimizing any data loss during the analysis. Additionally, we incorporated eXplainable Artificial Intelligence (XAI) techniques to investigate how EEG channels may interact distinctively for patients with ADHD, ADHD+CD, and CD. This underscores another innovative aspect of our study which we included as a new pointer in line 115 as:

- We employed the XAI technique (Grad-CAM) to visualize the interactions between EEG channels for patients with ADHD, ADHD+CD, and CD. This technique highlighted significant pairs of correlated EEG channels that played a crucial role in the classification of ADHD, ADHD+CD, and CD patients.

**Reviewer #2: The logic as well as the specific details of the information narrated in this article is much adequate, but I still have some minor issues:**

**1. it is recommended to attach demographic information for both private and public datasets, such as information on age, gender, and scales (if available).**

Thank you for your feedback. Regarding the internal private dataset, we furnished details regarding the age range (7–16 years old) and the clinician's utilization of the Diagnostic and Statistical Manual of Mental Disorders, Fourth Edition, Text Revision (DSM-IV-TR) diagnostic criteria for diagnosis. Unfortunately, gender information was not available for this dataset.

As for the external public dataset, we have provided DSM-IV as the diagnostic criteria and have newly included the age information in line 177 as: “This study's available EEG data comes from (Ali Motie Nasrabadi, Armin Allahverdy, Mehdi Samavati, 2020), which includes 61 children with ADHD and 60 healthy controls (HC), all within the age range of 7 to 12 years old.”. Regrettably, specific gender-related information for this dataset was also unavailable.

**2. I am concerned that this study used exact 21.25s as segment duration, is it possible to attach the experimental procedure for choosing this duration and is this time related to the paradigm design?**

Thank you for the comments. The segment duration of exactly 21.25 seconds was determined through an iterative hyperparameter tuning process. After conducting several trials and experiments, we found that a segment length of 21.25 seconds yielded the best performance. However, it's important to note that deep learning models are considered 'black box' models, making it challenging to precisely ascertain the reasons behind this optimal duration. Therefore, we arrived at this conclusion through multiple experimentation with varying segment durations. We also refrained from presenting results for other segment durations to maintain the clarity and focus of our findings, as including them might have potentially diluted the results or caused confusion for our readers. Nonetheless, we have included in line 171 for internal private dataset, that “The choice of a 21.25-second epoch duration was determined through extensive experimentation involving various segment durations. After thorough testing, it became evident that utilizing a 21.25-second epoch consistently produced the most optimal and favorable results.”

We also done the same for external public dataset in line 189: “Likewise, the utilization of a 4-second epoch duration was determined as optimal through thorough experimentation with various segment durations, consistently showcasing superior results.”

**3. How was the DL model structure designed? Why are the size and order of the convolutional and pooling layers arranged in this way? Have other deep learning models been considered? (page 210)**

Thank you for the comment. Similar to the previous comment, we engaged in an iterative hyperparameter tuning process to finalize the model's structure. Our objective was to ensure that the model's parameters were optimized to achieve the best performance. The key distinction from the previous comment lies in our approach: we initially established an initial model to identify the optimal segment duration. Subsequently, we fine-tuned the model parameters to further enhance performance. Likewise, we chose not to display results of our hyperparameter tuning process to uphold the clarity and coherence of our study. However, on line 222, we included additional clarification regarding the utilization of hyperparameters to validate and confirm the layer parameters of our model as: “After undergoing multiple rounds of hyperparameter tuning, we developed ADHD/CD-NET, illustrated in Fig. 3 and outlined in Table 2. This deep CNN model was specifically designed to classify channel-wise Continuous Wavelet Transform (CWT) correlation matrices into three distinct classes: ADHD, ADHD + CD, and CD.”

Additionally, we conducted experiments with various deep learning models, including a hybrid of CNN-LSTM, autoencoders, and transfer learning models. Despite exploring these alternatives, we ultimately opted for ADHD/CD-NET due to its simpler model architecture, resulting in lower computation requirements, and its ability to achieve higher classification accuracy simultaneously. We decided not to present the classification results of the other model, as the achieved classification accuracy was approximately 60%, which we deemed not worth reporting.
